# Long-term complications after stent assist coiling dependent on clopidogrel response

**DOI:** 10.1186/s12883-021-02270-0

**Published:** 2021-06-28

**Authors:** Kenji Shoda, Yukiko Enomoto, Yusuke Egashira, Takamasa Kinoshita, Daisuke Mizutani, Toru Iwama

**Affiliations:** Department of Neurosurgery, Gifu University Graduate School of Medicine, 1-1 Yanagido, Gifu, Gifu 501-1194 Japan

**Keywords:** Dual antiplatelet therapy, Stent assisted coiling, Hemorrhagic events, Chronic phase

## Abstract

**Background:**

Dual antiplatelet therapy (DAPT) is necessary for stent assisted coiling. However, long term use of DAPT has a potential risk of hemorrhagic events. We aimed to examine the relationship between clopidogrel reactivity and complications.

**Methods:**

Patients who underwent stent assisted coiling for unruptured aneurysms or previously treated aneurysms and received periprocedural DAPT in our institution between August 2011 to March 2020 were included. Platelet reactivity for clopidogrel was measured by VerifyNow assay system, and we defined the cut off value of P2Y12 Reaction Units (PRU) at 208 and classified patients as hypo-responders (PRU≧208) or responders (PRU<208). The rates of hemorrhagic and thrombotic events within 30 days (acute phase) and 30 days after the procedure (delayed phase) were compared between the two groups. Furthermore, changes in hemoglobin levels were measured before and after the procedure and at chronic stages (1 to 6 months thereafter).

**Results:**

From 61 patients included in this study, 36 patients were hypo-responders and 25 patients were responders. Hemorrhagic events occurred 8.0% only in responders in the acute phase (*p* = 0.16), and 2.78% in hypo-responders and 20.0% in responders in the delayed phase (*p* = 0.037). Changes in hemoglobin levels before and after the procedure were 1.22 g/dl in hypo-responders and 1.74 g/dl in responders (*p* = 0.032) while before the procedure and chronic stages they were 0.39 g/dl in hypo-responders and 1.39 g/dl in responders (*p* <  0.01). Thrombotic events were not significantly different between the two groups.

**Conclusion:**

Long term use of DAPT after stent assisted coiling is related to hemorrhagic events in the delayed phase. Preventing for hemorrhagic events, the duration of DAPT should be carefully considered in clopidogrel responders.

## Background

In the treatment of intracranial aneurysms, adjunctive techniques such as the balloon assist technique or stent assisted coiling are not only effective for wide-necked aneurysms but also decrease recurrence rates. Periprocedural dual antiplatelet therapy (DAPT) is necessary to reduce increasing thrombotic complications as the procedure complexity augments [1–3]. However, the duration of DAPT varied greatly among previous studies, and an optimal duration remains unknown [4–6], despite long-term DAPT use having a potential risk for hemorrhagic events. Before the procedure, measuring platelet reactivity is recommended, since platelet reactivity shows a high level of interindividual variability, particularly in the case of clopidogrel, which must be converted to a biologically active metabolite by cytochrome P450 enzymes (CYP) [7].

This study aimed to clarify the relationship between clopidogrel responses and complications. We measured platelet aggregation activity using VerifyNow (Accumetrics, San Diego, CA, USA) and investigated its association to clopidogrel responses and the occurrence of hemorrhagic and thrombotic events within 30 days of stent assisted coiling and 30 days after stent assisted coiling.

## Methods

### Study population

There were 77 aneurysms treated with stent assisted coiling for unruptured intracranial aneurysms (*n* = 67) and previously treated aneurysms (*n* = 10) using DAPT from August 2011 to March 2020 at our institution. Whole blood samples were obtained from patients at the time of the initial femoral artery puncture to measure platelet reactivity by VerifyNow. Patients who could not be measured platelet aggregation activity measurements by VerifyNow before the procedure (*n* = 9), and those who discontinued DAPT at least 1 month after the procedure (*n* = 7) were excluded. According to American College of Cardiology Foundation (ACCF)/ American Heart Association (AHA) 2011 guidelines [8], we defined the cut off value of P2Y12 Reaction Units (PRU) at 208^,^ and classified patients as hypo-responders (PRU≧208) or responders (PRU<208). We investigated hemorrhagic and thrombotic events within 30 days after the procedure (acute phase) and between one to 6 months after the procedure (delayed phase). Hemorrhagic events were defined as major bleeding events by the International Society of Thrombosis and Hemostasis [9], and thrombotic events were defined as symptomatic hyperintensity lesions on diffusion weighted imaging or transit ischemic attacks. In addition, we tracked changes in hemoglobin (Hb) levels from prior to the procedure to right afterwards and in chronic stages (time of hemorrhagic event or latest visit under DAPT treatment in the delayed phase). Demographic and clinical data collected included age, sex, smoking history, previously treated aneurysms, and the presence of comorbid hypertension, hyperlipidemia, diabetes mellitus, or chronic renal failure. This study was approved by the ethics committee of Gifu University Graduate School of Medicine (No. 2019–184). Due to the retrospective nature of the study, the need for informed written consent was waived by the ethics committee of Gifu University Graduate School of Medicine.

### Antiplatelet therapy

Antiplatelet drugs were started 7 days before the procedure, and these usually included clopidogrel 75 mg and aspirin 100 mg. On the morning of the procedure day, we measured platelet reactivity by Light Transmittance Aggregometry. And just before the procedure, final platelet reactivity was measured by VerifyNow. After inserting the sheath, we injected heparin aiming for an activated clotting time of 250–300 s. After the procedure, we initiated continuous intravenous administration of 10,000 units of heparin for 2 days. Six months after the procedure, we moved from DAPT to single antiplatelet therapy. However, if hemorrhagic events occurred, DAPT was discontinued from 1 to 3 months.

### Statistical analysis

All statistical analyses were performed using commercially available software (JUMP 14; SAS Institute, Cary, NC, USA). The student’s t-test was used for comparisons with continuous variables and the Fisher exact test for comparisons with categorical variables. Significant differences were defined as a *P* value *p* <  0.05.

## Result

**Fig. 1:**
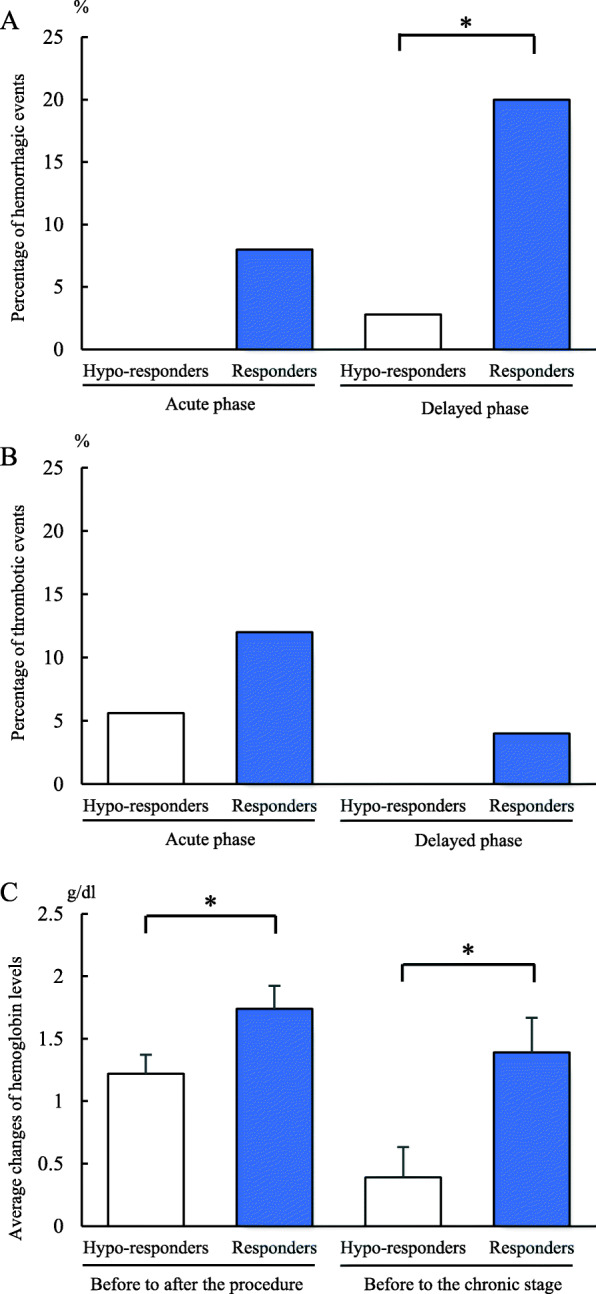
**A** shows the percentage of hemorrhagic events in the acute phase and the delayed phase, and **B** shows thrombotic events (hypo-responders: *n* = 36, responders: *n* = 25). **C** shows average changes of hemoglobin levels before the procedure to after and before the procedure to the chronic stage (hypo-responders: *n* = 26, responders: *n* = 21). Each column and bar represent the mean ± *SE*. All of them compared hypo-responders and responders. * = *p* < 0.05 by student’s t-test

Sixty-one patients were enrolled in this study. Of those, 36 were hypo-responders (mean PRU 262.4 ± 44.1) while 25 were responders (mean PRU 141.4 ± 42.3). Regarding changes in Hb levels, 14 patients did not have a blood test in our hospital during the chronic phase, therefore 47 patients were evaluated. (hypo-responders: *n* = 26, responders: *n* = 21) Demographic and clinical data and are shown in Table 1. There are no significant differences between the two groups. There were 21 Enterprise, 10 Neuroform, and 30 LVIS stents used in this study. The rate of hemorrhagic events in the acute phase was not significantly different between the two groups (*p* = 0.16) (Fig. 1A, Table 2). Two patients in the responders group suffered from gastrointestinal hemorrhage. Furthermore, hemorrhagic events in the delayed phase were more frequently observed in responders (*p* = 0.037) (Fig. 1A, Table 3). Thrombotic events occurred in two patients (5.6%) in the hypo-responders group and three patients (12.0%) in the responders group during the acute phase (*p* = 0.39) (Fig. 1B). Thrombotic events in the delayed phase occurred one patient in the hypo-responders group (Fig. 1B), and one patient in the hypo-responders group showed stent stenosis by follow up angiography 6 months after the procedure. The difference in Hb levels from before to after the procedure was higher in responders than in hypo-responders (1.74 g/dl vs 1.22 g/dl, *p* = 0.032), as was the change in Hb levels from before the procedure and the chronic stage (1.39 g/dl vs 0.39 g/dl, *p* < 0.01) (Fig. 1C, Table 4). Patients who were changed earlier to single antiplatelet therapy due to hemorrhagic events did not develop neither hemorrhagic nor thrombotic events in the delayed phase.

**Table 1:** Baseline demographics

	Hypo-responders (*n* = 36)	Responders (n = 25)	*p* value
Age (years)	62.4 ± 2.24	62.6 ± 2.38	0.97
Male sex (%)	10 (27.8)	7 (28.0)	1
PRU	262.4 ± 7.36	142.1 ± 8.59	< 0.01
ARU	489.1 ± 11.7	482.5 ± 16.1	0.73
Body mass index	22.5 ± 0.61	22.3 ± 0.61	0.85
Smoking (%)	9 (25.0)	6 (24.0)	1
Previously treated aneurysms (%)	5 (13.9)	5 (20.0)	0.73
Blood sample			
Hb (mg/dl)	13.1 ± 0.22	13.5 ± 0.29	0.31
Platelet (10^4^/μL)	21.9 ± 0.99	24.8 ± 1.27	0.08
PT-INR	0.94 ± 0.01	0.92 ± 0.01	0.42
comorbidities			
Hypertension (%)	19 (52.8)	20 (76.0)	0.11
Diabetes mellitus (%)	0	2 (8.0)	0.16
Hyperlipidemia (%)	15 (41.7)	17 (64.0)	0.12
Chronic renal failure (%)	5 (13.89)	3 (12.0)	1

Continuous variables are presented as average (mean ± *SE*). Categorical variables are presented as number of patients (percentage). *PRU* P2Y12 Reaction Units; *ARU* Aspirin Reaction Units; *Hb* Hemoglobin; *PT-INR* prothrombin time international normalized ratio

**Table 2:** Complications after stent assist coiling during the acute and delayed phases

Events		Hypo-responders (n = 36)	Responders (n = 25)	*p* value
Hemorrhagic events	Acute phase	0	2 (8.0%)	0.16
	Delayed phase	1 (2.8%)	5 (20.0%)	0.037
Thrombotic events	Acute phase	2 (5.6%)	3 (12.0%)	0.39
	Delayed phase	0	1 (4.0%)	0.41

**Table 3:** Hemorrhagic events in the delayed phase

Case	PRU	Response	Hemorrhagic event	Hb change (g/dl)	After the procedure
1	296	Hypo-responder	Gastrointestinal bleeding	5	2 months
2	94	Responder	Hematuria	2.2	4 months
3	123	Responder	Gastrointestinal bleeding	2.2	3 months
4	175	Responder	Intracranial hemorrhage	4.5	2 months
5	197	Responder	Genital bleeding	3.5	1 month
6	206	Responder	Hemorrhagic infarction	2.3	2 months

*PRU* P2Y12 Reaction Units; *Hb* hemoglobin

**Table 4:** Hb change from before until after the procedure and the chronic stage

Hb change	Hypo-responders (n = 26)	Responders (n = 21)	*p* value
Before to after the procedure (g/dl)	1.22 ± 0.15	1.74 ± 0.18	0.032
Before to chronic stage (g/dl)	0.39 ± 0.24	1.39 ± 0.28	< 0.01

Continuous variables are presented as average (mean ± SE). *Hb* hemoglobin.1

## Discussion

In our study, there was no difference in the incidence of thrombotic events between the two groups, although responders had hemorrhagic events at a significantly higher rate. In interventions for coronary arteries, the AHA/ American Stroke Association (ASA) guideline recommends patients to continue DAPT including P2Y12 inhibitors after stenting [10], and in the neuroendovascular treatment area, antiplatelet drugs are also employed for preventing thrombotic complications. However, the optimal duration of DAPT after stent assist coiling is uncertain. There are some reports on the timing and risk of thrombotic events, for instance, Matsumoto et al. reported that thrombotic events after the procedure are most likely to occur within 40 days after stent assisted coiling even if patients receive DAPT [11]. Furthermore, Song et al. reported that blood vessel tortuosity and large parent vessel size lead to incomplete stent apposition, becoming a risk factor of thrombotic complications [12].

Clopidogrel is an antiplatelet drug of widespread application. In Japan, it is administered in approximately 80% of cases in the perioperative period of neurovascular treatments [13]. Clopidogrel is a prodrug which needs to be converted into an active metabolite by CYP. Although CYP2C19 is the major enzyme involved in this process, its genetic variants can affect individual clopidogrel responses. Therefore, measuring platelet reactivity before the procedure is relevant. There are some methods to measure platelet functions, VerifyNow being one of the major points of care platelet reactivity analysis tools and employed on large clinical trials [14, 15]. Some studies have shown an association of hyper-responders to clopidogrel hemorrhagic events while hypo-responders are associated with thrombotic events, although a clear cutoff value has not been defined for neuroendovascular treatments and this value differed among studies [16–22]. A previous report involving the use of the Pipeline indicated that the ideal PRU value for avoiding both hemorrhagic and thrombotic events was between 70 and 150 [22]. Based on the ACCF/AHA 2011 guideline [8], we defined 208 as cutoff value and investigated hemorrhagic and thrombotic events both in the acute and delayed phase. In this study, the occurrence of thrombotic events was not significantly different between the groups, although hemorrhagic events frequently occurred in responders. In addition, Hb levels decreased more from before until after the procedure and during chronic stages in responders than in hypo-responders. Despite not meeting the criteria of International Society of Thrombosis and Hemostasis for major bleeding [9], some patients complained of subcutaneous bleeding, epistaxis, or hematuria, which could be associated with low Hb levels in the chronic stage.

Although some studies of stent assisted coiling reported an association of hemorrhagic and thrombotic events with PRU values in the acute phase [17–21], there are few studies of an association between platelet reactivity and these events in the delayed phase after stent assisted coiling. Goh et al. reported that evaluating hemorrhagic events up to 6 weeks after endovascular stenting treatment indicated that patients with > 72% PRU inhibition had more major bleeding than those with PRU < 72% [23]. Song et al. also referred to an association between clopidogrel response and thrombotic events in the delayed phase [21]. These authors reported that thrombotic events, symptomatic and asymptomatic ischemic stroke with positive findings on brain MRI in the territory of the treated aneurysm, or a transient ischemic attack more than 30 days after stent assisted coiling were not significantly correlated with PRU values. In agreement with this finding, our study showed a relationship between PRU values and hemorrhagic events, but not thrombotic events, in the delayed phase. Consequently, PRU appears to be more associated with hemorrhagic events in the delayed phase.

A previous study on DAPT duration in other treatment areas, reported that long-term DAPT use after ischemic stroke or after previous transient ischemic attacks increased the risk of major to life-threatening bleeding [24]. In addition, a recent study showed that, among patients undergoing coronary stents, 1 month of DAPT followed by clopidogrel single antiplatelet therapy resulted in a significantly lower rate of a composite of cardiovascular and bleeding events compared to 12 months of DAPT with clopidogrel and aspirin. Thus, a shorter DAPT duration after stenting may be more beneficial compared to long-term DAPT [25]. Even in cases of hemorrhagic events where DAPT was changed to single antiplatelet therapy 1 to 3 months before usual, there were no thrombotic events. Patients treated for aneurysm with neuro interventions are usually younger and thus, have fewer arteriosclerosis risk factors compared to patients treated with cerebrovascular or coronary revascularization [21], maybe reducing the risk of thrombotic events [23].

In our study, we found that clopidogrel responders had an increased risk of hemorrhagic events in the delayed phase, and PRU is useful for the prediction of hemorrhagic events. Although DAPT is necessary for thrombotic event prevention, hemorrhagic risk and DAPT duration should be carefully considered in clopidogrel responders. Further studies are needed to determine the ideal duration of DAPT administration.

### Limitations

There are several limitations to this study. First, this study is retrospective in nature, and hence subjected to potential confounding effects and bias. Second, we evaluated only 61 cases for postoperative complications. Therefore, it is quite possible that the statistical power of this study is insufficient to detect small differences. Third, it is known that clopidogrel response may be delayed conversion to clopidogrel hyper response [26]. We measured PRU only during the preoperative period, and thus, our evaluation may not be accurate.

## Conclusion

Our study demonstrates that long-term DAPT use after stent assisted coiling can lead to hemorrhagic events. Both hemorrhagic events in the delayed phase and decreased hemoglobin levels are associated with clopidogrel responses. Further, we showed that thrombotic events are not associated with PRU. DAPT duration should be carefully considered in clopidogrel responders.

## Data Availability

The datasets during the current study available from the corresponding author on reasonable request.

## Abbreviations

DAPT: Dual antiplatelet therapy
PRU: P2Y12 reaction units
CYP: Cytochrome P450 enzymes
ACCF: American College of Cardiology Foundation
AHA: American Heart Association
Hb: Hemoglobin
ASA: American Stroke Association
ARU: Aspirin reaction unit
PT-INR: Prothrombin time international normalized ratio
APTT: Activated partial thromboplastin time
